# Surgical Outcome and Malignant Risk Factors in Patients With Thyroid Nodule Classified as Bethesda Category III

**DOI:** 10.3389/fendo.2021.686849

**Published:** 2021-09-14

**Authors:** Jianhao Huang, Hongyan Shi, Muye Song, Jinan Liang, Zhiyuan Zhang, Xiaohang Chen, Yongchen Liu, Sanming Wang, Zeyu Wu

**Affiliations:** ^1^ Department of General Surgery, Guangdong Provincial People’s Hospital, Guangdong Academy of Medical Sciences, Guangzhou, China; ^2^ Shantou University Medical College, Shantou, China; ^3^ The Second School of Clinical Medicine, Southern Medical University, Guangzhou, China; ^4^ School of Medicine, South China University of Technology, Guangzhou, China

**Keywords:** thyroid cancer, autoimmune thyroid antibodies, tumor size, fine needle aspirate (FNA), microcalcification

## Abstract

**Background:**

Thyroid nodules are a very common finding in the general population. Fine-needle aspiration (FNA) has been recommended as the initial test for the evaluation of thyroid nodules. The trend on reporting as atypia of undetermined significance (AUS) has been significantly increased, but the malignant risk is still controversial among different studies. The aim of this study is to investigate the malignancy risk of thyroid nodules reported as Bethesda category III (AUS/FLUS) on initial FNA.

**Method:**

We reviewed 272 papillary thyroid cancer (PTC) patients with suspicious thyroid nodules who underwent fine-needle aspiration and received surgical treatment during 2019 to 2020.

**Results:**

One hundred ten (40.4%) patients were diagnosed with PTC. Multivariate analysis showed that microcalcification (p = 0.037, OR = 2.260, 95% CI: 1.051–4.860), shape (p = 0.003, OR = 4.367, 95% CI: 1.629–11.705), diameters (p = 0.002, OR = 0.278, 95% CI: 0.123–0.631), anti-thyroglobulin antibodies (TGAb) (p = 0.002, OR = 0.150, 95% CI: 0.046–0.494), anti-thyroid peroxidase antibody (A-TPO) (p = 0.009, OR = 4.784, 95% CI: 1.486–15.401), and nodule goiter (p < 0.001, OR = 0.100, 95% CI: 0.046–0.217) were independent malignant risk factors in patients with thyroid nodule classified as Bethesda category III.

**Conclusion:**

In this study, malignant risk factors in patients with thyroid nodule classified as Bethesda category III were significantly associated with preoperative serum TGAb, A-TPO, microcalcification, irregular shape, and nodule diameters. Nodules with malignant factors should be carefully elevated; surgery may be the better option for those patients.

## Introduction

Fine-needle aspiration (FNA) has been widely recommended as the initial test for the evaluation of thyroid nodules. For better communication of the results of FNA among clinicians, in 2007 The Bethesda System for Reporting Thyroid Cytopathology (TBSRTC) was established (1). In 2017, the TBSRTC updated for reclassifying some neoplasms as new categories and implying cancer risk for each category, which provide management recommendation for clinicians (2).

However, Bethesda category III (atypia of undetermined significance [AUS] or follicular lesion of undetermined significance [FLUS]) carries controversy because of its heterogeneity in different populations, institutions, and pathologists. The trend on AUS has been significantly increased after the implementation of the TBSRTC (3). For an AUS/FLUS nodule, in 2017 TBSRTC, it carried 10%–30% cancer risk with cytological or architectural atypia. In recent studies, the cancer risk of the AUS nodule varied from 31.2% to 46.5% (4–7), which are obviously higher compared to those in the Bethesda System. Therefore, category III with AUS/FLUS should be reconsidered to determine the true risk of malignancy and provide proper recommendation of the clinicians.

We focused our study on investigating the malignancy risk of thyroid nodules reported as Bethesda category III (AUS/FLUS) on initial FNA, combined with the results of preoperative ultrasound examination and several common laboratory indexes, such as preoperative serum Tg (thyroglobulin) and thyroid antibodies.

## Methods

### General Clinical Materials

Patients with suspicious thyroid nodules who underwent fine-needle aspiration and received surgical treatment in the Department of General Surgery at Guangdong Provincial People’s Hospital from January 2019 to December 2020 were enrolled in this study. The study was approved by the Research Ethics Committee of Guangdong Provincial People’s Hospital, Guangdong Academy of Medical Sciences. All patients gave their informed consent to the collection of data according to the local ethic committee indications. The preoperative US was routinely performed to assess the thyroid nodule status in all these patients. Ultrasonographic features of malignancy included hypoechoic nodule(s), undefined margin, irregular shape (taller than wide), microcalcifications, and capsule discontinuities. In the Laboratory Department, the levels of serum Tg (normal ranges: 3.5–77 ng/ml), TGAb (anti-thyroglobulin antibodies) (normal ranges: 0–115 IU/ml), A-TPO (anti-thyroid peroxidase antibody) (normal ranges: 0–34 IU/ml), and TRAb (thyrotropin receptor antibodies) (normal ranges: 0–1.75 IU/l) were measured before surgery. Each nodule received FNA three times, performed by experienced surgeons. Patients were diagnosed with malignancy by general pathological examination in the Pathology Department; the results were confirmed by two independent experienced pathologists.

Some patients were excluded from our study basing on any one of the following criteria: 1) mixed histologic types of thyroid malignancy; 2) received prior surgery or radiotherapy of the neck; 3) patients had proper surgical indications, including hyperthyroidism, retrosternal goiter; and 4) patients received active surveillance. Finally, the total number of 272 patients met our criteria.

### Statistical Analysis

Statistical analysis was performed by using IBM SPSS statistics 25.0 software. Univariate analyses by the X^2^ test or Fisher’s exact test were performed to investigate the malignant risk factors. Multivariate analysis was performed by binary logistic regression. p values <0.05 were considered statistically significant.

## Result

### Clinicopathological Characteristics of the Study Patients

The clinicopathological characteristics of the 272 study patients according to CLNM status are listed in **Table 1**. Among these, 110 (40.4%) patients were diagnosed with PTC (papillary thyroid cancer); 162 (59.6%) patients with benign nodules. The mean age was 43.8 ± 11.2. Two hundred four patients were female (75.0%), and 25.0% (68 patients) were male. One hundred seventy-seven (65.0%) patients had nodules less than 1 cm, while 95 (35.0%) patients had nodules larger than 1 cm. With the FNA pathologic result, a total of 263 (96.7%%) patients had nucleus deformity, including obvious nucleoli and large nuclei. One hundred thirty-nine (51.1%) patients had irregular cell arrangement. Nucleus grooves were found in 142 patients (52.2%), while intranuclear pseudoinclusions were observed in 39 patients (14.3%). One hundred twenty-nine (46.0%) patients had nodular goiter.

**Table 1:** Clinicopathological characteristics and univariate analysis of thyroid nodules (n = 272).

	Benign (n = 162)	Malignant (n = 110)	p-value
Gender			0.203
Male	36	32	
Female	126	78	
Age			0.123
<55	132	98	
≥55	30	12	
Diameters of nodule			0.000*
≤1 cm	84	93	
>1 cm	78	17	
Location of nodule			0.357
Upper lobe	18	17	
Others	144	93	
Hashimoto’s thyroiditis			0.254
Yes	45	23	
No	117	87	
Nodule			
Goiter			0.049*
Yes	105	20	
No	57	90	
Marked hypoechoic			0.000*
Yes	87	18	
No	75	92	
Margin			0.000*
Well-defined	87	14	
Non-well-defined	75	96	
Shape			0.000*
Regular	99	16	
Irregular	63	94	
Microcalcification			0.000*
Yes	69	76	
No	93	34	
Nucleus deformity			0.743
Yes	156	107	
No	6	3	
Cell arrangement			0.267
Yes	78	61	
No	84	49	
Nucleus Grooves			0.002*
Yes	72	70	
No	90	40	
Intranuclear pseudoinclusions			0.078
Yes	18	21	
No	144	89	
Tg			
≤77 ng/ml	150	99	0.508
>77 ng/ml	12	11	
TGAb			0.04*
≤115 IU/ml	105	89	
>115 IU/ml	57	21	
TRAb			0.096
≤1.75 IU/ml	159	103	
>1.75 IU/ml	3	7	
TPO			0.002*
≤34 IU/l	138	75	
>34 IU/l	24	34	

*Statistically significant.

### Malignant Risk Factors in Patients With Thyroid Nodule Classified as Bethesda Category III

In the univariate analyses, as shown in **Table 2**, we found that malignant diagnosis in patients with thyroid nodules classified as Bethesda category III was significantly related to nodule diameter (p < 0.001), nodule goiter (p = 0.049), marked hypoechoicity (p < 0.001), margin (p < 0.001), shape (p < 0.001), microcalcification (p < 0.001), level of TGAb (p = 0.04), and A-TPO (p = 0.002), and nucleus grooves (p = 0.002). There were no significant differences in other clinicopathological factors such as age, intranuclear pseudoinclusions, Tg, or TRAb. For the multivariate analysis, a binary logistic regression was performed, and it revealed that microcalcification (p = 0.037, OR = 2.260, 95% CI: 1.051–4.860), shape (p = 0.003, OR = 4.367, 95% CI: 1.629–11.705), diameters (p = 0.002, OR = 0.278, 95% CI: 0.123–0.631), TGAb (p = 0.002, OR = 0.150, 95% CI: 0.046–0.494), A-TPO (p = 0.009, OR = 4.784, 95% CI: 1.486–15.401), and nodule goiter (p < 0.001, OR = 0.100, 95% CI: 0.046–0.217) were independent malignant risk factors in patients with thyroid nodules classified as Bethesda category III, as shown in **Table 2**.

**Table 2:** Multivariate analysis of the malignant risk factors of thyroid nodules.

	Sig.	OR (95% CI)
Grooves	0.171	1.631 (0.810–3.285)
Microcalcification	0.037*	2.260 (1.051–4.860)
Shape	0.003*	4.367 (1.629–11.705)
Margin	0.399	1.545 (0.562–4.252)
Marked hypoechoic	0.801	0.880 (0.325–2.383)
Nodule goiter	0.000*	0.100 (0.046–0.217)
Diameters	0.002*	0.278 (0.123–0.631)
A-TPO	0.009*	4.784 (1.486–15.401)
TGAb	0.002*	0.150 (0.046–0.494)

*Statistically significant.

## Discussion

According to 2017 TBSRTC, the risk of malignancy for these Bethesda III thyroid nodules is estimated to be 10%–30%, but recent studies have reported malignancy rates significantly above the predicted ratio (2). In our retrospective study, the malignancy ratio was considerably higher, reaching 40.4% of all thyroid nodules. Ho et al. (8) presented 37.8% malignant risk which was determined based on the AUS/FLUS nodules undergoing surgery after receiving FNA, which is similar with our study. Gweon et al. (9) reported overall risk of malignancy for initial Bethesda III thyroid nodules with relatively high 55.5%. Besides, those nodules without repeat FNA show 78.3% malignant rate, significantly different from the malignant rate that received repeat FNA (37.2%). For the management of Bethesda class III nodules, repeat FNA may be recommended. However, Lee et al. (10) demonstrated that the usage of repeat FNA should be limited because of the possible unavoidable difference in the cytological interpretation. Similarly, Ogmen et al. (11) pointed out that repeat biopsy of AUS/FLUS nodules may not enhance the diagnosis of malignancy. In other studies, the risk of malignancy was found to be 31.2%–46.5%, again higher compared to the proposed one in the Bethesda System (4–7). On the contrary, Nagarkatti et al. (12) found that among 125 AUS/FLUS patients directly receiving surgery, the malignant rate was 16.0%, similar with the estimated risk rate according to 2017 TBSRTC.

Several studies have closely investigated the clinical features in the AUS/FLUS nodule to determine whether patients should receive direct surgery (6, 13). Ultrasonography was the fundamental evaluation of thyroid nodules because it can distinguish the suspicious malignant nodules that are suitable for undergoing FNA (14). In our study, we found that microcalcification and irregular shape (taller than wide) were malignant risk factors by multivariate analysis in ultrasound examination. These ultrasound features were generally considered most likely related to malignancy. In a meta-analysis including 1,851 nodules with indeterminate cytology aspirates, the presence of microcalcifications showed the best specificity (96%) (15). This finding is consistent with our results regarding microcalcifications as predictor of malignancy, with OR of 2.260 on multivariate analysis. Irregular shape (taller than wide) was also usually contributed to predict the malignancy of the intermediate thyroid nodule (14, 16, 17). Nodule size is also reported to have predictive value of malignancy in several studies (8, 18), and Jung et al. (6) suggest that size <2 cm is associated with an increased risk of well-differentiated thyroid cancer, which is also supported by Hadi et al. (19) However, Miller et al. (20) and Karman et al. (21) demonstrated that size >2 cm may be associated with thyroid malignancy. In our study, we found that nodule size in the malignant group is significantly smaller than that in the benign group, and on the multivariate analysis this factor was confirmed to be an independent predictor for malignancy and a threshold of approximately 1 cm in nodule diameter. FNA in thyroid nodules with diameter ≤1 cm is little studied, but we assumed that those with malignant ultrasound features should be carefully elevated and received FNA.

Another finding was that normal TGAb was more likely to be diagnosed as PTC than elevated TGAb (p = 0.008, OR = 3.493, with 95% CI: ranging from 1.378–8.861). TGAb, defined as thyroglobulin antibody, is a major thyroid-specific protein and mistakenly attacks healthy organs and tissues in autoimmune diseases. TGAb was widely used in the diagnosis of autoimmune thyroid diseases: Hashimoto disease, postpartum thyroiditis, neonatal hypothyroidism, and Grave’s disease. However, whether preoperative serum TGAb could predict the malignancy is still controversial (22).

Several studies discovered that autoimmune thyroiditis is associated with thyroid carcinoma, while others have proposed that the coexistence of thyroiditis in patients with thyroid nodules confers no additional risk of malignancy (23, 24). Besides, even in the absence of the histologically confirmed autoimmune thyroiditis, some studies underlined that preoperative serum TGAb was at a higher level in Differentiated Thyroid Cancer (DTC) patients than in those with benign thyroid nodules, and it was an independent predictive factor of DTC (22, 25). In our study, the incidence of malignancy in TGAb ≤115 IU/ml and TGAb >115 IU/ml was 45.9% *vs.* 26.9%, with a p value <0.05 in multivariate analysis, which may indicate that the presence of the autoimmune thyroiditis may interfere the malignant diagnosis of the intermediate thyroid nodules. MacDonald et al. (26) recognized that hyperplastic follicular cells on FNA samples from HT may mimic a follicular neoplasm, leading to false-positive results. Follicular cell changes in HT may be mistaken for thyroid neoplasm resulting in false-positives (27). We assume that patients had intermediate thyroid nodules and coexist with elevated TGAb which should have additional examinations to determine the malignancy, such as PET-CT and repeat FNA.

A-TPO, in our study, was significantly correlated with the malignancy of the nodule classified as Bethesda category III (p = 0.009, OR = 4.784, 95% CI: 1.486–15.401). A-TPO, defined as thyroid peroxidase antibody, is the antibody against TPO which plays an important role in the production of thyroid hormones. The presence of A-TPO may indicate thyroid damage by the autoimmune system, which may induce the malignant change of the thyroid nodule. The existence of A-TPO confers the risk of PTC with thyroid nodules, demonstrated by Wu et al. (28). However, other few studies may not identify the relationship between the level of A-TPO and the malignancy risk of the thyroid nodules (29, 30).

In addition, our study had a relatively small sample size, which may limit the statistical power of subgroup analysis. Moreover, our study was only a single-center retrospective analysis. It needs to be further verified by multicenter prospective studies.

## Conclusions

In this study, malignant risk factors in patients with thyroid nodule classified as Bethesda category III was significantly associated with preoperative serum TGAb and A-TPO, which were little studied and still controversial in previous studies. The other independent predictors were microcalcification, irregular shape, and nodule diameters. Nodules with diameter ≤1 cm with malignant ultrasound features should be carefully elevated and receive FNA.

Thus, these factors are worth being considered by the surgeons when evaluating the thyroid nodule classified as Bethesda category III. Larger multicenter studies are necessary to be carried out to help clinicians build a strategy toward thyroid nodule classified as Bethesda category III.

## Data Availability Statement

The original contributions presented in the study are included in the article/supplementary material. Further inquiries can be directed to the corresponding author.

## Ethics Statement

The study was approved by the Research Ethics Committee of Guangdong Provincial People’s Hospital, Guangdong Academy of Medical Sciences. All patients gave their written informed consent to the collection of data according to the Research Ethics Committee of Guangdong Provincial People’s Hospital, Guangdong Academy of Medical Sciences.

## Author Contributions

Conception and design: JH, MS, and HS. Provision of study materials or patients: YL, SW, and ZW. Collection and assembly of database: JH, MS, HS, JL, ZZ, and XC. Data analysis: JH, MS, and HS. All authors contributed to the article and approved the submitted version.

## Funding

This work was supported by Guangdong Basic and Applied Basic Research Foundation (No. 2020A1515010126), Scientific Research Staring Foundation for the Returned Overseas from Guangdong Provincial People’s Hospital (No. 2017x02) and Guangdong Provincial People’s Hospital Scientific Foundation for Distinguished Young Scholars of Guangdong Province (No. KJ012019441).

## Conflict of Interest

The authors declare that the research was conducted in the absence of any commercial or financial relationships that could be construed as a potential conflict of interest.

## Publisher’s Note

All claims expressed in this article are solely those of the authors and do not necessarily represent those of their affiliated organizations, or those of the publisher, the editors and the reviewers. Any product that may be evaluated in this article, or claim that may be made by its manufacturer, is not guaranteed or endorsed by the publisher.
