# Corrigendum: Surgical outcome and malignant risk factors in patients with thyroid nodule classified as Bethesda category III

**DOI:** 10.3389/fendo.2024.1517690

**Published:** 2024-11-15

**Authors:** Jianhao Huang, Hongyan Shi, Muye Song, Jinan Liang, Zhiyuan Zhang, Xiaohang Chen, Yongchen Liu, Sanming Wang, Zeyu Wu

**Affiliations:** ^1^ Department of General Surgery, Guangdong Provincial People’s Hospital, Guangdong Academy of Medical Sciences, Guangzhou, China; ^2^ Shantou University Medical College, Shantou, China; ^3^ The Second School of Clinical Medicine, Southern Medical University, Guangzhou, China; ^4^ School of Medicine, South China University of Technology, Guangzhou, China

**Keywords:** thyroid cancer, autoimmune thyroid antibodies, tumor size, fine needle aspirate (FNA), microcalcification

In the published article, there was an error in the Funding statement. The Funding statement for the Guangdong Basic and Applied Basic Research Foundation was displayed as “Natural Science Foundation of Guangdong Province (No. 2020A1515010127)”. The correct statement is “Guangdong Basic and Applied Basic Research Foundation (No. 2020A1515010126)”. The correct Funding statement appears below.

“This work was supported by Guangdong Basic and Applied Basic Research Foundation (No. 2020A1515010126), Scientific Research Staring Foundation for the Returned Overseas from Guangdong Provincial People’s Hospital (No. 2017x02) and Guangdong Provincial People’s Hospital Scientific Foundation for Distinguished Young Scholars of Guangdong Province (No. KJ012019441).”

The authors apologize for this error and state that this does not change the scientific conclusions of the article in any way. The original article has been updated.

